# MR‐Linac‐guided stereotactic radiotherapy for CT‐indiscernible intravascular renal cell carcinoma tumours

**DOI:** 10.1002/bco2.428

**Published:** 2024-08-30

**Authors:** Mihir D. Shanker, Zhiqian Henry Yu, Jinzhong Yang, Surena Matin, Matthew T. Campbell, Pavlos Msaouel, Nizar Tannir, Surendra Prajapati, Yao Ding, Belinda Lee, Angela Sobremonte, Chad Tang

**Affiliations:** ^1^ The University of Texas MD Anderson Cancer Centre Houston Texas USA; ^2^ The University of Queensland Brisbane Australia

## INTRODUCTION

Inferior vena cava tumour thrombus (IVC‐TT) is a life‐threatening complication of advanced renal cell carcinoma (RCC) occurring in 10%–25% of patients with RCC with one third of patients having concurrent distant metastatic disease.^1^, ^2^ Surgical resection in the form of radical nephrectomy and caval thrombectomy is the established option for obtaining local control of the disease and is associated with long‐term oncologic control; however, only 50% of patients are operative candidates at time of diagnosis.^3^, ^4^ Untreated RCC IVC‐TT has a poor natural history, with a median survival of 5 months with a 1‐year disease‐specific survival of 29%.^5^ Stereotactic ablative body radiotherapy (SBRT) is a potentially feasible and safe option in patients who are not surgical candidates with the potential to be used for a wide range of RCC indications, from definitive local control in inoperable candidates, advanced‐stage disease palliation and to improve survival outcomes in oligometastatic settings. However, renal disease‐restricted contrast use and poor visualization of RCC IVC‐TT with conventional computed tomography (CT) impairs precise planning and treatment delivery. Magnetic resonance linear accelerator (MR‐Linac)‐based therapy is a novel technology, which allows advanced visualization of thrombi targets for improved delineation, inter‐ and intra‐fraction monitoring and adaptive treatment.

We report the world‐first use of this approach in five patients treated at a major North American cancer centre. Patient, tumour, and treatment characteristics, toxicity, and local control outcomes of IVC‐TT patients treated with a 1.5 T MR‐Linac at the University of Texas MD Anderson Cancer Center were retrospectively evaluated. Patients received 40–50 Gy in five fractions over 2 weeks. Planning tumour volume (PTV) (uncertainty) margins were 3–7 mm anisotropically. Plans were generated in Monaco treatment planning system (v5.40, Elekta Solutions AB, Stockholm, Sweden). Primary outcomes were local control, radiological response as per the Response Evaluation Criteria in Solid Tumours (RECIST) 1.1 through follow‐up imaging, composite clinical/biochemical palliation outcomes and toxicity.

**FIGURE 1:**
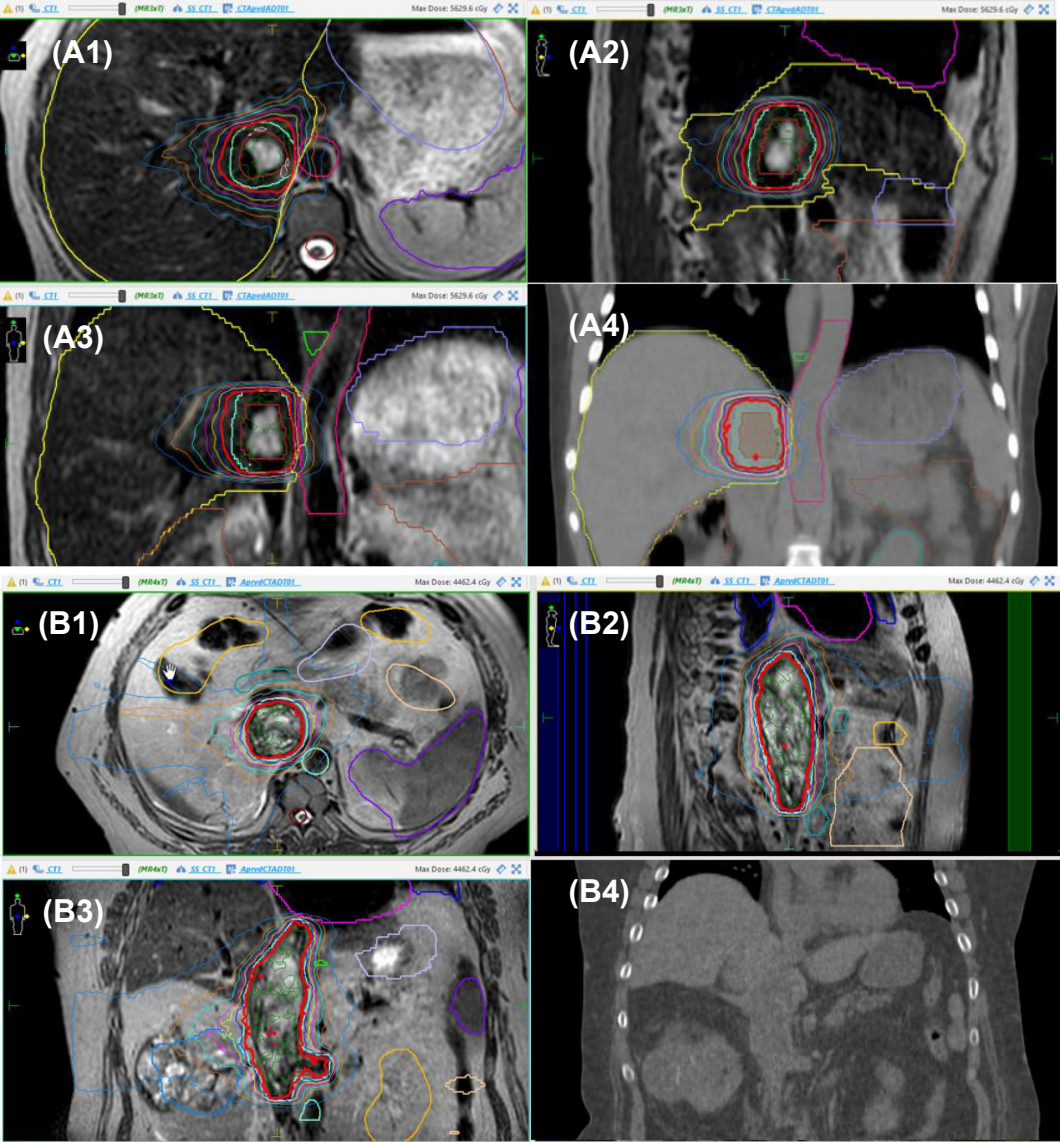
(A1–A3) and (B1–B3) Real‐time visualization of inferior vena cava (IVC) tumour thrombus on magnetic resonance linear accelerator (MR‐Linac) with respective dosimetry. (A4) Comparison of coronal section of dosimetry superimposed onto CT highlighting inability to discern tumour thrombus target. (B4) Comparison of visualization on standard simulation CT imaging demonstrating poor discrimination of IVC tumour target.

The median age was 52 years (interquartile range [IQR] 45–56), and median follow‐up was 11.1 months (IQR 8.9–12.2). Sixty percent of patients had a Mayo Level III IVC, with two patients having a level IV extension. The median tumour volume was 12.8 cc (IQR 4.4–22.9). One patient had symptomatic lower limb edema and abdominal pain, and one other patient had deranged liver function parameters prior to treatment. During treatment planning, visualization of the tumour‐IVC interface was indiscernible with standard diagnostic non‐contrast CT but visualized with MR‐based simulation and during treatment using MR guidance, which permitted radiotherapy dose escalation to maximize local control (Figure 1). Intrafraction visualization additionally highlighted asymmetrical motion of the IVC‐TT target during normal respiratory motion, with a greater movement at the superior infrahepatic region compared to the inferior infrahepatic region. All initial plans met target and normal tissue dose constraints. One of the five patients required adjusting the target, duodenum, and bowel bag on two of the five fractions. One patient died 8.9 months post treatment due to systemic progression. A summary of patient characteristics is presented in Table S1.

At the time of the last follow‐up, no patients had local progression, and no adverse clinical or biochemical toxicity events were associated with SBRT. Four patients demonstrated a radiological response, and one patient had stable disease. One patient demonstrating deranged liver‐function‐tests (LFTs) 4× upper limit of normal (ULN) at baseline had a biochemical improvement to 2× ULN at the end of the 2‐week treatment course and complete normalization 1‐month post‐SBRT. One patient experiencing abdominal pain and lower limb edema experienced complete resolution 3 weeks post completion of treatment. Two case study examples of MR‐Linac guided therapy are presented in Figure 1.

Untreated IVC‐TT has a poor natural history with cause of death primarily due to cardiac failure, pulmonary embolism or Budd‐Chiari syndrome.^6^ Tumour thrombi demonstrate concomitant organized tumour cells within the fibrin clot matrix, which promotes a higher degree of thrombogenicity compared to bland thrombi alone. Anticoagulation, whilst effective for bland thrombus in reducing embolic risk, will not sterilize malignant cells, and as such, definitive local control strategies are required to mitigate clinical sequelae of untreated IVT‐TT.^7^ This study demonstrates that MR‐Linac‐guided SBRT is a safe and promising strategy, which can be considered in the armamentarium of treatment options for RCC IVC‐TT in addition to surgery and minimally invasive techniques.^8^ There were no local failures in the cohort and no clinical or biochemical toxicity events. All patients with clinical or biochemical abnormalities prior to treatment achieved resolution within 4 weeks of completion of SBRT. MR‐Linac‐guided SBRT enabled daily visualization of tumours that would have been indiscernible with standard non‐contrast CT. Real‐time on‐treatment MR visualization elucidated changes in daily tumour position, shape and changes in adjacent sensitive organs such as bowel and duodenum, which would have not been visualized during conventional radiotherapy treatment delivery. During daily treatment, this advanced visualization permitted adaptive planning with the ability to modify the radiotherapy treatment plan to comply with real‐time changes in both the target IVC‐TT and adjacent organ position changes. Superior MR‐based visualization both during treatment planning and delivery in turn permits a reduction in uncertainty margins and concomitantly allows safe dose escalation to maximize tumour control and minimize toxicity outcomes. The magnitude of intrafraction target motion due to respiration is summarized in Table S2. Institutional organ‐at‐risk constraints over five fractions are summarized in Table S3.

Dose escalation to ablative doses is challenging with conventional CT‐based planning and poor‐quality on‐treatment imaging with conebeam CT‐based delivery. Resultingly, patients unsuitable to operative management are limited to palliative doses of radiotherapy with large radiotherapy fields, which limit effective long‐term local control. In this study, excellent local control was attained through the safe delivery of dose‐escalated radiotherapy despite the use of narrow margins of 3–7 mm. Asymmetrical margins were utilized based on the elucidated asymmetrical motion of the IVC‐TT target during normal respiratory motion, a physiological characteristic not previously known during conventional radiotherapy delivery. Adaptive planning was permitted for enhanced fractional target coverage and decreased normal tissue dose, particularly to adjacent bowel. Whilst this study is limited by its small sample size and overall short duration of follow‐up, the initial signal for local control at a median of 11.1 months is highly promising in this high‐risk population and warrants longer follow‐up and outcomes in a greater number of patients.

This letter reports outcomes and presents MR‐Linac‐guided SBRT as a novel and promising treatment strategy, which can be considered in the armamentarium of treatment options for definitive local control in inoperable patients or for palliation of symptoms and/or biochemical derangements secondary to RCC IVC‐TT. The technology enabled daily visualization of tumours that would have been indiscernible with standard conebeam CT‐based daily image guidance, and the ability to visualize the intravascular tumour thrombus during simulation, planning and treatment delivery enabled safe radiotherapy dose escalation to maximize local control outcomes. Further prospective studies with larger patient populations and a longer duration of follow‐up will further characterize the value of this treatment strategy in the overall management of inoperable IVC‐TT.

## CONFLICT OF INTEREST STATEMENT

The authors declare no conflicts of interest.

## Supporting information


**Table S1.** Summary of patient characteristics.

**Table S2.** Intrafraction target motion due to respiration based on review of cine images composed of 3 imaging planes (axial, sagittal, and coronal planes) passing through the geometric center of the GTV. Image resolution = 1.136mmx1.136mm.

**Table S3.** Normal tissue constraints (5 fractions) for MRI‐guided adapative radiotherapy for RCC IVC‐TT.
