# Supplementary material for: MR‐Linac‐guided stereotactic radiotherapy for CT‐indiscernible intravascular renal cell carcinoma tumours
Source: BJUI Compass. 2024 Aug 30;5(10):913–5. doi: 10.1002/bco2.428 (PMC11479802; doi:10.1002/bco2.428)
Supplement: Supplementary file 1 — Table S1. Summary of patient characteristics. [file BCO2-5-913-s003.docx]

|  | **Age** | **Dose / fractionation** | **Biologically equivalent dose (BED) (Gy) (α/β=2.6)** | **GTV  volume (cc)** | **Mayo classification** | **Histological subtype** | **Metastatic disease at treatment** | **Systemic therapy prior to SBRT** | **Concurrent systemic therapy with SBRT** | **Prior nephrectomy** | **Surgery following SBRT** | **Radiological response (RECIST 1.1)** | **Pre-SBRT clinical/biochemical abnormalities** | **Post-SBRT clinical/biochemical improvement** |
| --- | --- | --- | --- | --- | --- | --- | --- | --- | --- | --- | --- | --- | --- | --- |
| **Patient 1** | 52 | 5 Fx (40Gy/5) | 163.08 | 1.85 | III | Clear cell | Yes | Yes | No | Yes | Yes | Partial | Clinical: None  Biochemical: Elevated ALT, AST, Alkaline phosphatase, and lipase. | Clinical: N/A  Biochemical: Reduction of deranged LFTs by 80% noted by completion of final fraction of treatment. Complete resolution to baseline 1 month post SBRT completion |
| **Patient 2** | 56 | 5 Fx (40Gy/5) | 163.08 | 180.15 | IV | Clear cell | Yes | Yes | Yes (Cabozantinib) | Yes | No | Partial | Clinical: None  Biochemical: None | N/A |
| **Patient 3** | 45 | 5 Fx (50Gy/5) | 242.3 | 12.8 | III | Papillary | No | No | No | Yes | No | Partial | Clinical: None  Biochemical: None | N/A |
| **Patient 4** | 20 | 5 Fx (40Gy/5) | 163.08 | 22.93 | III | MiT translocation type | Yes | Yes | Yes (Cabozantinib) | No | No | Partial | Clinical: Lower limb edema, right upper quadrant abdominal pain  Biochemical: None | Clinical: Resolution of lower limb edema and abdominal pain 3 weeks post completion of SBRT.  Biochemical: N/A |
| **Patient 5** | 62 | 5 Fx (40Gy/5) | 163.08 | 4.378 | IV | Clear Cell | Yes | No | No | Yes | No | Stable | Clinical: None  Biochemical: None | N/A |

Supplementary Table 1: Summary of patient characteristics
