# Supplementary material for: MR‐Linac‐guided stereotactic radiotherapy for CT‐indiscernible intravascular renal cell carcinoma tumours
Source: BJUI Compass. 2024 Aug 30;5(10):913–5. doi: 10.1002/bco2.428 (PMC11479802; doi:10.1002/bco2.428)
Supplement: Supplementary file 2 — Table S2. Intrafraction target motion due to respiration based on review of cine images composed of 3 imaging planes (axial, sagittal, and coronal planes) passing through the geometric center of the GTV. Image resolution = 1.136mmx1.136mm. [file BCO2-5-913-s002.docx]

Supplementary Table 2: Intrafraction target motion due to respiration based on review of cine images composed of 3 imaging planes (axial, sagittal, and coronal planes) passing through the geometric center of the GTV. Image resolution = 1.136mmx1.136mm.

| **Cases** | **LR motion (X) (mm)** | | **AP motion (Y) (mm)** | | | **SI motion (Z) (mm)** | | |  |
| --- | --- | --- | --- | --- | --- | --- | --- | --- | --- |
|  | **Mean** | **STD** | **Mean** | **STD** | | **Mean** | **STD** | |  |
| Patient 1 | <1 |  | 1.47 | 0.17 | | 6.35 | 0.45 | |  |
| Patient 2 | <1 |  | 1.83 | | 0.25 | 6.39 | | 0.62 | |
| Patient 3 | <1 |  | 2.5 | | 0.3 | 5.62 | | 0.59 | |
| Patient 4 | <1 |  | 1.88 | | 0.45 | 4.46 | | 0.94 | |
| Patient 5 | <1 |  | 2.19 | | 0.2 | 6.33 | | 0.43 | |

Abbreviations: LR – left-right; AP – anterior-posterior; SI – superior-inferior; STD: standard deviation
