# Supplementary material for: MR‐Linac‐guided stereotactic radiotherapy for CT‐indiscernible intravascular renal cell carcinoma tumours
Source: BJUI Compass. 2024 Aug 30;5(10):913–5. doi: 10.1002/bco2.428 (PMC11479802; doi:10.1002/bco2.428)
Supplement: Supplementary file 3 — Table S3. Normal tissue constraints (5 fractions) for MRI‐guided adapative radiotherapy for RCC IVC‐TT. [file BCO2-5-913-s001.docx]

| Supplementary Table 3 : Normal tissue constraints (5 fractions)​ for MRI-guided adapative radiotherapy for RCC IVC-TT | |
| --- | --- |
| Duodenum​ | Dmax < 30-35 Gy, D5cc < 18 Gy, D10cc < 12.5Gy​ |
| Small bowel​ | Dmax < 28 Gy​ |
| Large Bowel​ | Dmax < 30-35 Gy, V30 < 1 cc, V25 < 10 cc, V15 < 30 cc​ |
| Stomach​ | Dmax < 28-35Gy, V30 < 1 cc, V25 < 10 cc, V15 < 30 cc​ |
| Bowel bag​ | Dmax < 28 Gy​ |
| Liver ​ | V12 <50%​ |
| Liver - GTV​ | 700 cc < 15 Gy, Mean < 16 Gy​ |
| Left kidney​ | Dmax < 10 Gy​ |
| Right kidney​ | V10<50%​ |
| Esophagus​ | Dmax < 35 Gy, D5cc < 19.5 Gy​ |
| Heart​ | V40 < 10%, Dmax < 42 Gy​ |
| Aorta​ | V40<1cc, Dmax <56Gy​ |
| Common bile duct​ | Dmax < 55 Gy​ |
| Total lung​ | mean dose ≤ 6 Gy, V5 ≤ 30%, V10 ≤ 17%, V20 ≤ 12%, ​ |
| Right lung​ | mean dose ≤ 10 Gy, V10 ≤ 35%, V20 ≤ 25%, V30 ≤ 15%​ |
| Spinal cord​ | Dmax < 18-23Gy, V10cc < 15 Gy, V20<0.5-1cc​ |

Abbreviations: MRI-Magnetic resonance imaging, GTV-Gross tumour volume, IVC-TT: Inferior vena cavae tumour thrombus, RCC: Renal cell carcinoma.
